# Supplementary material for: Updates on the Proposed Botulinum Toxin A Mechanisms of Action in Orofacial Pain: A Review of Animal Studies
Source: Toxins (Basel). 2025 Nov 23;17(12):567. doi: 10.3390/toxins17120567 (PMC12737382; doi:10.3390/toxins17120567)
Supplement: Supplementary file 1 [file toxins-17-00567-s001.zip › toxins-3922505-supplementary.pdf]

toxins

Review

Supplementary Materials: Updates on the Proposed Botulinum Toxin A Mechanisms of Action in Orofacial Pain: A Review of Animal Studies

Jaime Fabillar Jr., Yumiko Yamamoto, Kazuyuki Koike, Daisuke Ikutame and Yoshizo Matsuka

Risk of Bias Evaluation of Included Studies

The SYRCLE-RoB assessment identified considerable variability in the methodological quality of the included studies (Figure S1).

- *Selection bias:* The majority of studies failed to report procedures for random sequence generation or allocation concealment, leading to assessments of "unclear risk."
- *Performance and detection bias:* Blinding of personnel and outcome assessors was rarely documented. Although several studies referenced the use of standardized laboratory conditions, explicit statements regarding blinding were generally absent.
- *Attrition bias:* Most studies reported complete outcome data and were therefore rated as low risk in this domain.
- *Reporting bias:* All studies presented outcomes as specified in their methods; nonetheless, the lack of pre-registration contributed to several "unclear risk" ratings.
- *Other biases:* Common issues involved insufficient reporting of sample size calculations, exclusive use of single-sex cohorts (predominantly male), and inconsistencies in dosing regimens.

In summary, the included studies exhibited moderate overall methodological quality, with persistent concerns regarding incomplete reporting, particularly in the domains of randomization and blinding. These methodological limitations should be carefully considered when interpreting mechanistic findings derived from animal models.

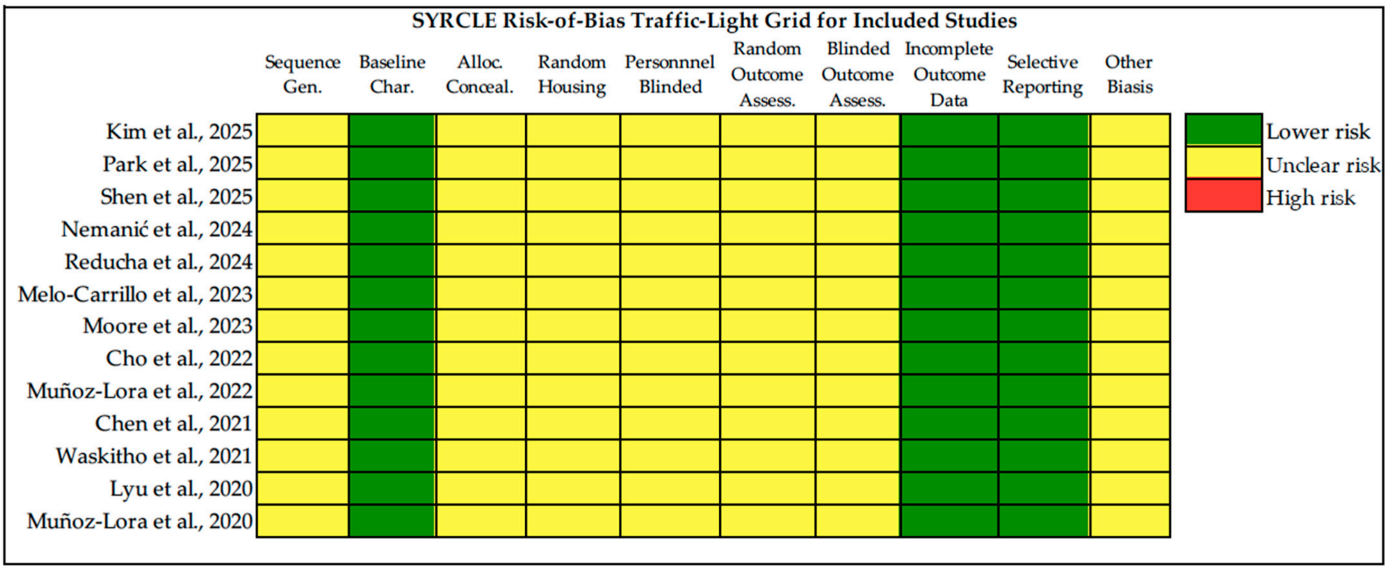

Figure S1. SYRCLE risk-of-bias traffic-light assessment for included animal studies.
This figure presents the methodological quality assessment of the thirteen included animal studies, evaluated using the SYRCLE Risk of Bias tool. Each cell denotes the risk classification for a specific domain within an individual study, illustrated with a standardized color scheme (green: low risk; yellow: unclear risk; red: high risk). Most studies were rated as having a low risk of bias in terms of baseline characteristics, incomplete outcome data, and selective reporting. Conversely, the majority of other domains, such as sequence generation, allocation concealment, random housing, blinding of personnel, and blinding of outcome assessors, were predominantly rated as unclear risk due to insufficient reporting.

Disclaimer/Publisher's Note: The statements, opinions and data contained in all publications are solely those of the individual author(s) and contributor(s) and not of MDPI and/or the editor(s). MDPI and/or the editor(s) disclaim responsibility for any injury to people or property resulting from any ideas, methods, instructions or products referred to in the content.
